# Anxiety disorders in ancient Indian literature

**DOI:** 10.4103/0019-5545.71009

**Published:** 2010

**Authors:** Hitesh C. Sheth, Zindadil Gandhi, G. K. Vankar

**Affiliations:** Department of Psychiatry, Hospital for Mental Health, Vikas Gruh Road, Jamnagar, Gujarat, India; 1 Department of Psychiatry, Albany Medical College, New York; 2 Department of Psychiatry, B.J Medical College, Ahmedabad, India

**Keywords:** Ancient epics, anxiety disorders, generalized anxiety disorder, Iliad, post traumatic stress disorder, Ramayana, Shrimad Bhagavatam

## Abstract

In western literature, the oldest description of symptoms of PTSD, an anxiety group of disorder, is seen in Homer’s Iliad written around 720 BC. According to Shay, Achilles was suffering from symptoms of PTSD. However, in the Indian literature it was mentioned around 5000 BC. The description of a PTSD-like syndrome is seen in the *Ramayana*, although it was not described as PTSD or by any other similar name. Ravana’s brother Marrich was having symptoms of PTSD after he was grievously hurt by Lord Rama’s arrow and was almost dead. This traumatic event threatened his physical integrity. He developed all the symptoms of PTSD, like hyper-arousal, re-experiencing the events and avoidance. He also gave up his natural work of harassing the monk and got engaged in meditation and austerities. His symptoms lasted for many years till Lord Rama killed him, while he was masquerading as a golden deer to deceive Sita. In another ancient epic *Shrimad Bhagavatam*, Maharshi Ved Vyasa described the symptoms of Generalized Anxiety Disorder (GAD). The demon King Kansha developed GAD-like symptoms, when Lord Krishna killed all his demons and threatened to kill him. He developed symptoms of GAD, like excessive worry about the attack from his arch foe Krishna, difficulty in concentration and difficulty in falling asleep. Like Marrich, the symptoms of Kansha also lasted until Lord Krishna killed him.

## INTRODUCTION

The post traumatic stress disorder (PTSD), which is one of the anxiety disorders codified in DSM-3 in 1980, has become subject of intense debate about its nature and origins. The earliest description of PTSD-like symptoms in the Western literature is seen in Homer’s Iliad. In Achilles in Vietnam Shay presents the Iliad as the tale of Achilles’ tragedy and as a description of men at war. Shay in the first five chapters of his book looks carefully at Achilles’ tribulations. Achilles was betrayed by his commander Agamemnon. The betrayal is described as shirking “Achilles’ social and moral horizon.” As a result of this, Achilles is portrayed as driven by rage, caring only about a small group of combat- proven cohorts. This is followed by the loss of his foster brother, Patroklos, in battle. Patroklos was also his second in command and closest friend. This leads to grief and suicidal ruminations. Achilles is portrayed as guilt-ridden with survivor’s syndrome, as bereft of his will to live and as feeling dead already. Ultimately, Achilles is found to go berserk, committing atrocities on both living and dead.[1] This, for Shay, is the story of the *Iliad* and also as Shay goes on to demonstrate, the story of many Vietnam combat veterans.[2] The approximate year of creation of *Iliad* is 720 BC.[3] But according to other researchers PTSD always existed in past but was unrecognized by contemporaries.[4] The symptoms of PTSD were also seen in a family trapped in the Bergemoleccto avalanche in mid-18^th^ century.[5] According to some researchers PTSD existed in ancient times but the emphasis was more on somatic symptoms and less on psychological symptoms like flashbacks. Flashbacks, a dissociative state, are defined as involuntary images that occur in waking stage.[6]

In recent times, PTSD was first described as ‘Irritable Heart’ by Jacob D’Costa in 1871. In World War I, it was called as ‘Shell Shock’. In World War II, it was known as ‘Combat Neurosis’. However after vietnam war, psychiatric co-morbidity of associated war veterans brought the concept of Post Traumatic Stress Disorder, as we know now. This is the earliest description of PTSD seen in Western literature. However, in the Indian literature the great sage Valmiki mentioned it five thousand years back (5114 BC) in the epic *Ramayana*.[7] Although Valmiki described symptoms similar to PTSD, he didn’t describe it as a syndrome or a disease.

The description of symptoms of generalized anxiety disorder, the commonest of the anxiety group of disorder, is seen in the great epic ‘Shrimad Bhagavatam’ written by Maharshi Vedvyasa in 900 AD.[8]

## DISCUSSION

### Lord Rama, demon Marrich and post traumatic stress disorder

The earliest description of the symptoms of PTSD is seen in our greatest and oldest epic *Ramayana*. It was written by Maharshi Valmiki in the Sanskrit language and later Goswami Tulsidas wrote the same epic in the Hindi language, though Goswami Tulsidas didn’t describe symptoms as explicitly as Maharishi Valmiki.

The demon Marrich, a cousin of Ravana, was suffering from PTSD-like symptoms. Marrich had the strength of one thousand elephants. Like all demons (demons don’t have horns on their head, they just get perverted happiness in harassing other people) he used to derive pleasure from sadistic activities. He used to harass the rishis by throwing bones in their sacrificial fire, obstructing their Yagna (ritual rites) and sometimes killing them and drinking their blood. Marrich was the same demon that deceived Sita by masquerading as a golden deer and thus helped Ravana in her abduction.

Rishi Vishwamitra had brought Rama and Laxamana from Ayodhya to protect his yagna. When Marrich was trying to enter into Rishi Vishwamitra’s Ashram to destroy his yagna, Rama saw Marrich trespassing the ashram’s territory, so he challenged him for a fight. However, Marrich ignored Rama, thinking him as a mere boy because at that time Rama was only 16 years old. Rama killed the other demons accompanying Marrich and also hit him with a blunt arrow that threw him in the sea, a hundred miles far from the shore. When Marrich regained consciousness after a long time, he found himself in the sea. Then Marrich, stunned by the attack, got up and went to Lanka (Verse: 17, 18, 19, 21, 22, Chapter 38, Aranyakand, Valmiki Ramayana).[9]

Now let us compare the symptoms of Marrich with the symptoms of PTSD mentioned in DSM IV criteria[10] (American Psychiatric Association, 1994). According to DSM-IV criteria: After experiencing or witnessing a traumatic event the symptoms of re-experiencing, avoidance and hyper-arousal should be present for more than one month and should lead to impairment in social and occupational functioning.

Many years after that traumatic incident, when Ravana asked Marrich to help him abduct Rama’s wife Sita, he stared blankly at Ravana. His mouth became dry on hearing the name of Rama. He began to lick his lower lip with his tongue. His heart was filled with terror and he began to tremble. He almost became unconscious (Verse: 22, 23, 24, Chapter 36 Aranyakand, Valmiki Ramayana),[9] (symptoms of hyper-arousal). He described Rama’s strength to Ravana and forbade him to abduct Sita. He said, “I know Rama’s strength. It is not prudent to fight against him. If you wish, you can fight against him alone but don’t involve me in that fight. If you want to see me alive, don’t discuss Rama in front of me.” He also described his own symptoms that he developed after Rama’s arrow hit him (Verse: 19, 20, Chapter 39, Aranyakand, Valmiki Ramayana).[9] He said, “Now, in every tree, I see Rama with bow and arrow, wearing black deer skin. Lord Rama appears to me like the God of Death (Yama). Sometimes I see thousands of Rama and I am filled with terror (Verse: 15, Chapter 39 Aranyakand, Valmiki Ramayana).[9] When I sit in solitude, I see nothing but Rama. Sometimes I see Rama in my dreams and I often lose consciousness. Sometimes I think that Rama is pervading the whole universe.” (Verse: 16, 17, and 18, Chapter 39, Valmiki Ramayana).[9] In Goswami Tulsidas’s ‘*Ramcharit Manas*’, Marrich says to Ravana, “I see Rama and his brother Laxmana everywhere. Both are extremely valiant and cannot be defeated in war.” (Verse 24, Aranyakand, Ramcharit Manas), (symptoms of re-experiencing). He further adds, “I am unable to hear the name of things which starts with the letter ‘R’. When I hear that, I begin to tremble, because the letter ‘R’ reminds me of Rama. So I avoid Rath (chariot), Ratna (gems) and other things that begin with ‘R’. (symptoms of avoidance) (Verse 18, Aranyakand, Valmiki Ramayana).[9]

Marrich’s symptoms lasted for many years (duration was more than one month). Marrich gave up his day-to-day activity of harassing Rishis and, got himself engaged in a meditation and austerities. Ravana also accused Marrich of being possessed by a ghost because he was refusing to help him to abduct Sita. At last, out of fear of Ravana, when Marrich acquiesced to help Ravana, only then Ravana thought that Marrich was free from the ghost. So the symptoms of re-experiencing traumatic events, avoidance and hyper-arousal were seen in Marrich after Rama threatened his physical integrity. Duration of symptoms was more than one month. According to Ravana, Marrich was having social impairment because he was not performing his natural duty of harassing monks and was living in solitude to meditate.

### Lord Krishna, Demon Kansha and generalized anxiety disorder

Maharshi Ved Vyasa in ‘*Shrimad Bhagavatam*’ described symptoms similar to GAD in demon king Kansha. Kansha, the king of Mathura, used to harass rishis engaged in askesis. During the time of his sister Devaki’s wedding, he heard a prophecy that Devaki’s eighth son would kill him. So, he imprisoned Devaki and her husband Vasudev. He also killed her first seven children. Her eighth son, Krishna was born in a prison but was safely sent to Vraj, where Nanda reared him.

Sage Narada wanted to see the early end of demon Kansha, by arranging his early confrontation with Krishna. So he informed Kansha that “Krishna; the eighth son of Devaki was alive and was living in Vraj.” Kansha sent demons to kill all newborn babies in Vraj. However, Lord Krishna killed all demons sent by Kansha. Learning about that news, Kansha got extremely angry (Verse 17-18, Chapter 36, *Shrimad Bhagavatam*).[12] Kansha, then sent two more demons Kesi and Vyomasura. Lord Krishna killed them too. Hearing that news, Kansha was terrified as he recalled the prophecy that the eighth son of Devaki would kill him. He perceived the threat to his life by Lord Krishna. He began to worry excessively about Krishna and became so restless that he thought of him while eating, drinking, sleeping, walking, talking and even while breathing (Verse 34, Chapter 44, *Shrimad Bhagavatam*).[12] As he was constantly petrified by the thoughts of his enemy, he must have had difficulty in concentrating on the tasks at hand. He used to see messengers of the lord of death (Yamadoots) while awake and also during his sleep, indicating that his end was near (Verse 26-27, Chapter 43, *Shrimad Bhagavatam*).[12] He was so much afraid of Krishna that he was unable to sleep during night. As Kansha was constantly thinking of his enemy without getting any sleep, he must have had difficulty in ruling his kingdom. His symptoms, similar to GAD, described in DSM IV, lasted for many years until he was killed by Lord Krishna. However, as he was always thinking of Lord Krishna, (it doesn’t matter whether one remembers God with enmity or devotion) he got nirvana!

Thus, both demon Marrich and demon king Kansha suffered from symptoms of anxiety disorders. However, their anxiety disorders were of a unique kind wherein both were having symptoms due to the incarnations of the lord. They were always thinking of God. The Lord killed both of them and both got Nirvana (salvation), which is difficult to accomplish even by performance of the great ascesis.

## CONCLUSION

An immeasurably vast and inscrutable sea of ancient Indian literature is littered with invaluable gems of knowledge and priceless pearls of wisdom and is eagerly awaiting researchers and scholars who can dive deep into its fathomless heart to collect them and exhibit them to the modern world. In Western literature the first description of symptoms of anxiety disorder is seen in Homer’s *Iliad* written around 720 BC. However, in the Indian literature description of symptoms similar to that of anxiety disorder is seen in our greatest epic *Ramayana* written by Maharshi Valmiki around 5000 BC. Later, Maharshi Ved Vyasa in *Shrimad Bhagavatam* also described the symptoms of anxiety disorder.
